# Barriers and facilitators to office-based opioid agonist therapy prescribing and effective interventions to increase provider prescribing: protocol for a systematic review

**DOI:** 10.1186/s13643-019-1076-7

**Published:** 2019-07-25

**Authors:** Lara L. Nixon, Jazmin C. Marlinga, K. Alix Hayden, Kelly J. Mrklas

**Affiliations:** 1 Department of Family Medicine, Cumming School of Medicine, University of Calgary, Room G012 3330 Hospital Drive NW, Calgary, AB T2N 4N1 Canada; 2 Libraries & Cultural Resources, University of Calgary, 2500 University Drive NW, Calgary, AB T2N 4N1 Canada; 3 Department of Community Health Sciences, Cumming School of Medicine, University of Calgary, 3330 Hospital Drive NW, Calgary, AB T2N 4N1 Canada; 4 Strategic Clinical Networks™, System Innovation and Programs, Alberta Health Services, 403 - 29th Street NW, Calgary, AB T2N 2T9 Canada

**Keywords:** Opioid agonist therapy, Methadone, Buprenorphine-suboxone, Primary care, Family physician, Prescribing, Barrier, Facilitator, Opioid use disorder, Opiate addiction

## Abstract

**Background:**

Opiate agonist therapy (OAT) prescribing rates by family physicians are low in the context of community-based, comprehensive primary care. Understanding the factors that support and/or inhibit OAT prescribing within primary care is needed. Our study objectives are to identify and synthesize documented barriers to, and facilitators of, primary care opioid agonist prescribing, and effective strategies to inform intervention planning and support increased primary care OAT prescribing.

**Methods/design:**

We will systematically search EMBASE, CINAHL, PsycINFO, Cochrane Central Register of Controlled Trials, MEDLINE, and gray literature in three domains: primary care providers, opioid agonist therapy, and opioid abuse. We will retain and assess primary studies reporting documented participation, or self-reported willingness to participate, in OAT prescribing; and/or at least one determinant of OAT prescribing; and/or strategies to address determinants of OAT prescribing from the perspective of primary care providers in comprehensive, community-based practice settings. There will be no restrictions on study design or publication date. Studies limited to specialty clinics with specialist prescribers, lacking extractable data, or in languages other than English or French will be excluded. Two reviewers will perform abstract review and data extraction independently. We will assess the quality of included studies using the Joanna Briggs Institute Critical Appraisal Tool. We will use a framework method of analysis to deductively code barriers and facilitators and to characterize effective strategies to support prescribing using a combined, modified a priori framework comprising the Theoretical Domains Framework and the Consolidated Framework for Implementation Research.

**Discussion:**

To date, no synthesis has been undertaken of the barriers and facilitators or effective interventions promoting OAT prescribing by primary care clinicians in community-based comprehensive care settings. Enacting change in physician behaviors, community-based programming, and health services is complex and best informed by using theoretical frameworks that allow the analysis of the available data to assist in designing and implementing interventions. In light of the current opioid crisis, increasing the capacity of primary care clinicians to provide OAT is an important strategy to curb morbidity and mortality from opioid use disorder.

**Systematic review registration:**

PROSPERO CRD86835

**Electronic supplementary material:**

The online version of this article (10.1186/s13643-019-1076-7) contains supplementary material, which is available to authorized users.

## Background

Of the pharmacotherapies available for opioid use disorder (OUD), two opiate-agonist agents, methadone and the combination therapy buprenorphine-naloxone, have been found to be effective in retaining patients in treatment and decreasing illicit opiate use [1, 2]. Both treatments are long-acting agonists that function at the level of the mu-opioid receptor to block the physical symptoms of opioid withdrawal and are designed to have minimal euphoric effects and block the euphoria associated with administering exogenous prescribed or illicit opioids [3]. Compared to abstinence alone in individuals with OUD, these agents reduce all-cause and overdose mortality and relapse to disordered patterns of opioid use and increase retention in addiction treatment programs [4–6]. In pregnant patients with OUD, both methadone and buprenorphine-naloxone improved neonatal outcomes with decreased rates of prematurity, lower neonatal abstinence scores, and decreased length of hospital stay, with higher treatment retention rates and decreased relapse rates during pregnancy [7, 8]. With improvements in disease outcomes, patients with OUD also benefit from improved social outcomes, including decreased rates of incarceration and increased rates of employment and improved social functioning [9].

Despite these benefits, patients with OUD experience many barriers to accessing methadone and buprenorphine-naloxone. Stigma and lack of timely access to treatment have often been cited as deterrents to patients receiving care [10–12]. Geographic differences in treatment availability add difficulty for patients outside of urban centers [13]. High-level barriers, such as financial coverage for therapies and regulatory restrictions, as well as institutional and provider-based attitudes, limit access to care [14]. Allied health team members similarly report challenges around providing opiate agonist therapy (OAT), with pharmacy services requiring additional supports [15] and nursing practices being affected by moral distress in the care of patients with addictions [16–18]. Patients with OUD have expressed a preference for receiving OAT from their family physicians who can provide comprehensive care and relational continuity for physical and mental health issues [19]. While it is often asserted that community-based family physicians are well positioned to prescribe OAT, a number of patient- and practice-specific factors must be considered to incorporate addiction care into general practice [20].

Initial data indicate that family physicians can successfully integrate medication-assisted treatment for OUD into comprehensive care [21, 22]. Despite this, methadone and buprenorphine-naloxone prescribing rates by family physicians remain low [23]. Studies have documented barriers to medication-assisted OUD treatment provision from a generalist perspective, often citing patient-, physician-, clinic-, and systems-based factors [24–30]. However, in preliminary searching, we were unable to identify a synthesis describing factors that enable and/or hinder the use of medication-assisted OAT by family physicians.

Implementing clinical innovations is challenging and requires complex changes in clinical practice, better collaboration between disciplines, and/or changes in organizations [31]. Prescribing opioid agonists in the context of community-based, comprehensive primary care may require a change in all three of these (or additional) domains (e.g., regular and special patient monitoring, close communication with pharmacists, and development of on-call coverage by appropriately licensed and knowledgeable teams). Identifying potential barriers to change and strategies to promote change is enhanced by exploration informed by different theoretical perspectives [31]. Two frameworks integrating multiple theories relating to behavioral change are the Theoretical Domains Framework (TDF) [32–34] and the Consolidated Framework for Implementation Research (CFIR) [35]. TDF is an evidence-based framework derived from 33 theories of behavior change and 128 constructs organized into 12 “theoretical domains” that focus on individual- and group-level determinants of change [33, 34]. CFIR consolidates 19 theories of implementation and consists of 5 domains (intervention characteristics, outer setting, inner setting, characteristics of individuals, and implementation process characteristics) complementary to TDF in their ability to systematically identify contextual, process, intervention, and individual characteristics of implementation.

This synthesis will use a modified combination of the TDF and CFIR frameworks to identify barriers to, facilitators of, and effective interventions for opioid agonist prescribing in comprehensive community-based care at multiple levels [36, 37]. The purpose for combining the frameworks is several-fold: first, TDF offers relative advantages in assessing individual- and group-level change factors compared to the CFIR; second, the CFIR offers relative advantages compared to the TDF in its ability to identify and describe in detail factors associated with context, interventions, and implementation processes; third, both frameworks are flexible and able to provide multi-level conceptualization; and fourth, the comprehensive theoretical perspective of the TDF offers researchers the opportunity to readily synthesize findings for optimized and rapid use with similarly structured intervention mapping techniques [38]. By combining the frameworks and modifying them slightly (e.g., subdomain merges to avoid overlapping codes in areas such as individual characteristics and environmental context/resources), we hope to provide the most comprehensive conceptual scope for coding, create important precursor knowledge for intervention design and mapping, and increase the usability of the evidence generated [36].

Although there is documented evidence for barriers, facilitators, and effective, provider-targeted interventions to increase opioid agonist prescribing in community-based comprehensive primary care, this body of evidence has not been synthesized in a form that is actionable using evidence-based, theory-driven intervention design and testing. The aim of this systematic review is to identify and synthesize available literature regarding barriers, facilitators, and effective strategies for primary care prescribing of OAT. The intent is to inform intervention planning and create ready-to-use evidence to encourage increased provider and policymaker use of this evidence-based addiction treatment practice.

## Methods

### Research objectives

The specific objectives of this study are to:

1. Locate the literature reporting barriers, facilitators, and effective strategies for primary care prescribing of OAT.
2. Extract reported barriers, facilitators, and effective strategies for primary care OAT prescription and categorize these according to TDF/CFIR domains.
3. Identify key themes within TDF/CFIR domains to inform the planning of future interventions to support primary care prescribing of OAT.

### Study design

Protocol design, search strategy, synthesis, and reporting of findings from this systematic review will be guided by the Sampling Strategy-Type of Study-Approaches-Range of Years-Limits-Inclusion and Exclusions-Terms used-Electronic Sources (STARLITE) Search Reporting Standards [39], the Cochrane Collaboration Handbook of Systematic Reviews [40], The Centre of Reviews and Dissemination (CRD) guide [41], the Preferred Reporting Items for Systematic Review and Meta-Analysis (PRISMA) Statement [42] and Protocols Extension (PRISMA-P) [43], and the Enhancing Transparency in Reporting the Synthesis of Qualitative Research (ENTREQ) reporting guidelines [44] (see Additional file 1). The synthesis will be guided by the Knowledge-to-Action (KTA) cycle [45–47], an overarching meta-framework that identifies the multiple, dynamic, and interacting phases involved in knowledge creation and knowledge application in practice. Systematic study of barriers and facilitators to knowledge use is a critical part of the “Action” cycle, and a key precursor to intervention design and implementation. Systematic study of interventions to support the application of knowledge (in this case OAT prescribing) documents effective strategies and can inform the design, tailoring, and testing of interventions, as well as help to overcome identified barriers to knowledge use. These steps are core parts of engineering strategies that encourage the uptake and use of opioid agonist prescribing in primary care. Planned action theory underpins the KTA cycle, which is flexible and allows for the easy integration of other theory-based approaches to behavior change, including the TDF and CFIR.

### Study eligibility criteria

#### Context

For the purposes of this study, we will define primary care as practice including comprehensive community-based continuous care focused on prevention and health promotion, as well as acute, chronic, and terminal illness management [48–51]. Reference to using pharmacologic treatment for managing heroin and opiate-dependent patients using opioid-agonists dates back to the early 1970s; methadone was approved by the United States Food and Drug Administration (US FDA) in 1972 for this indication, and sublingual buprenorphine-naloxone was approved by the US FDA on October 8, 2002; as buprenorphine has been available for much longer in some European countries, we will not date-limit included studies [52–56].

For the purposes of this systematic review, the term barrier will refer to any single- or multi-level factor negatively associated with, or that hinders the likelihood of, office-based primary care prescribing of opioid agonists. The term facilitator refers to any single- or multi-level factor positively associated with, or that increases the likelihood of, office-based primary care prescribing of opioid agonists [57]. Effective strategies to increase provider prescription of OAT are defined as any single- or multi-level intervention that reduces barriers or leverages facilitators and increases the likelihood of office-based primary care prescribing of opioid agonists (adapted from Mrklas et al. [58]).  

#### Participants

We will include studies that involve primary care physicians or nurse practitioners (providers) in a full scope setting or comprehensive primary care (e.g., scope of practice not limited to addiction treatment). Further, we will include studies that report documented barriers to, facilitators of, and/or effective interventions in which providers participate in, or self-report willingness to participate in, any of the following: (1) OAT prescribing, and/or (2) at least one determinant of OAT prescribing, and/or (3) strategies to address determinants of OAT prescribing, from the perspective of primary care providers in comprehensive community-based practice settings.

We will exclude studies involving only specialty clinics or providers (e.g., addiction clinics or psychiatric clinics focused on addiction treatment), studies reporting secondary data (e.g., scoping or systematic review articles), studies that lack extractable data, and studies in any language other than French or English. Reviews will be excluded, but their bibliographies will be used to identify potential citations. No restrictions will be placed on publication date, study design, or geographic location.

#### Outcomes

The primary study outcomes will be documented barriers, facilitators, and effective strategies to increase primary care OAT prescribing from the perspective of primary care providers in comprehensive community-based practice settings. Documented determinants of opioid agonist prescribing may include multi-level factors, including those related to the individual prescriber, OAT and its implementation, and the practice and system setting. Some of the factors identified in the literature include the following: lack of comfort with managing patients with opioid dependence; concerns that patients will divert their opioid agonist; lack of experience, training, or education in using opioid agonists; provider fear of practice shifting to patients with opioid dependence; fear of staff or community resistance; burdensome licensing requirements to prescribe opioid agonists; inadequate community support services (e.g., addiction counselling); and inadequate reimbursement [59].

We will extract information about effective strategies in a structured, inductive, and iterative way, starting from outcomes previously reported in the literature (e.g., publication of opioid agonist prescribing best practice guidelines oriented to primary care prescribers [60]; increasing addiction training in undergraduate, graduate, and continuing medical education [59]; and clarifying regulatory conditions for prescribing opioid agonists) [24]. Given the known importance of contextual factors in practice patterns [28, 29], multiple study characteristics will be iteratively abstracted (e.g., setting, community size, practice type, and licensing requirements for OAT prescribing) (Table 1).

**Table 1:** Draft data extraction tool

Variable name	Description
Study characteristics	
Author	Authors of publication
Year	Publication year
Country	Country of origin
Language	Language of publication
Funding source	Reported source of funding for the study
Population	Study population
Setting	Study setting
Study type	Study design
Community size	Rural urban or remote
Location of graduation	Location prescriber graduated from
Practice type	Private, group, solo, community health center
Licensing	Prescriber licensing to prescribe opioid agonists
Training	Training in addiction medicine or opioid agonist therapy
Training type	Type of training received in addiction medicine or opioid agonist therapy
Training type description	Description of training as reported by authors
Sample	Sample population
Study method	Study method used
Study participants	Number of study participants
Participant response rate	What proportion of participants responded
Gender	Reported gender of participants
Inclusion criteria	Reported inclusion criteria
Exclusion criteria	Reported exclusion criteria
Statistical analytic technique	Statistical technique(s) used in analysis
Documented Barriers and Facilitators	
TDF domains -Barriers or facilitators that reflect individual or group level influences on implementation will be coded to one of Michie et al 2005, TDF Domains (CFIR Domains II, III, and V will be used in lieu of TDF’s Environmental context and resources/Environmental constraints)	Knowledge Skills Social/professional role and identity Beliefs about capabilities Beliefs about consequences Motivation and goals Memory, attention, decision processes Social influences Emotion Behavioral regulation Nature of the behaviors
CFIR domains -Barriers-facilitators that reflect context will be coded into one of Damschroder et al. 2009 CFIR Domains (and their respective subdomains), excluding Domain IV – Individual Characteristics, which will be replaced by TDF domains	I. Intervention characteristics (8) II. Outer setting (4) III. Inner setting (5) V. Implementation process characteristics (4)
Outcomes	
Self-reported willingness to participate in prescribing office-based OAT	Self-reported willingness to prescribe office-based OAT
Actual participation in office-based OAT prescribing	Reported participation in office-based OAT
Actual participation in at least one determinant of office-based OAT prescribing	Reported participation in at least one determinant of OAT prescribing
OAT determinants	Reported determinants
Documented strategies (These strategies will be inductively analyzed in an iterative fashion, and coded as themes arise)	Reported strategies for enhancing provider prescription of OAT, or elements of OAT

### Data sources and search strategy

The search strategy design was informed by, and will be executed with, the guidance of an academic librarian (KAH). Preliminary searching (Cochrane Database of Systematic Reviews, Campbell Collaboration, Joanna Briggs Institute EBP Database, PROSPERO, and MEDLINE) was unsuccessful in identifying an existing synthesis of the documented barriers and facilitators of, and effective interventions for, primary care provider OAT prescribing in the context of comprehensive community-based primary care.

Several key papers were used to identify appropriate Medical Subject Headings (MeSH terms) and keywords and develop the initial search strategy for feasibility testing in MEDLINE (OVID). As the search strategy prioritized sensitivity (i.e., comprehensiveness), the search incorporates three main concepts: primary care provider, opioid substitution treatment, and opioid addiction. The MEDLINE strategy was circulated for peer review (DL) using the PRESS Checklist [61–63] and feedback integrated to create a final search strategy (Table 2). The MEDLINE search strategy will be adapted and translated for each database.

**Table 2:** Draft search strategy (MEDLINE)

#	Searches
1	exp Physicians, Primary Care/
2	exp General Practitioners/
3	exp Family Practice/
4	exp Physicians, Family/
5	exp Practice Patterns, Physicians’/
6	exp Nurse Practitioners/
7	exp Nurse Clinicians/
8	exp Primary Health Care/
9	exp Ambulatory Care/
10	exp Community Health Services/
11	exp Community Medicine/
12	exp Office Visits/
13	physician*.mp.
14	primary care physician*.mp.
15	family physician*.mp.
16	family doctor*.mp.
17	(general adj2 physician*).mp.
18	(general adj2 practitioner*).mp.
19	(family adj2 practitioner*).mp.
20	(primary care adj2 practitioner*).mp.
21	(nurse* adj2 clinician*).mp.
22	(nurse adj2 practitioner*).mp.
23	family practice*.mp.
24	family medicine.mp.
25	general practice*.mp.
26	outpatient practice*.mp.
27	primary care.mp.
28	primary care setting*.mp.
29	public sector healthcare.mp.
30	office-based.mp.
31	(private adj2 office*).mp.
32	(physician* adj2 office*).mp.
33	(office* adj2 visit*).mp.
34	(community adj3 care).mp.
35	(community adj3 healthcare).mp.
36	(community adj3 treatment).mp.
37	or/1-36
38	exp Opiate Substitution Treatment/
39	37 and 38
40	exp BUPRENORPHINE/
41	exp BUPRENORPHINE, NALOXONE DRUG COMBINATION/
42	exp Naloxone/
43	exp METHADONE/
44	(opioid adj2 treatment).mp.
45	(opioid adj2 therap*).mp.
46	(opioid adj2 substitution).mp.
47	(opioid adj2 replacement).mp.
48	(opioid adj2 maintenance).mp.
49	(opiate adj2 treatment).mp.
50	(opiate adj2 therap*).mp.
51	(opiate adj2 substitution).mp.
52	(opiate adj2 replacement).mp.
53	methadone.mp.
54	(methadone adj2 treatment).mp.
55	(methadone adj2 therap*).mp.
56	(methadone adj2 substitution).mp.
57	(methadone adj2 replacement).mp.
58	Buprenorphine.mp.
59	(buprenorphine adj2 treatment).mp.
60	(buprenorphine adj2 therap*).mp.
61	(buprenorphine adj2 substitution).mp.
62	(buprenorphine adj2 replacement).mp.
63	naloxone.mp.
64	(naloxone adj2 treatment).mp.
65	(naloxone adj2 therap*).mp.
66	(naloxone adj2 substitution).mp.
67	(naloxone adj2 replacement).mp.
68	(buprenorphine naloxone adj2 treatment).mp.
69	(buprenorphine naloxone adj2 therap*).mp.
70	(buprenorphine naloxone adj2 substitution).mp.
71	(buprenorphine naloxone adj2 replacement).mp.
72	(medication assisted adj2 treatment).mp.
73	(medication assisted adj2 therap*).mp.
74	(maintenance adj2 treatment).mp.
75	(maintenance adj2 therap*).mp.
76	(substitution adj2 therap*).mp.
77	opioid agonist*.mp.
78	or/40-77
79	exp Opioid-Related Disorders/
80	exp Heroin Dependence/
81	exp Morphine Dependence/
82	exp Opium Dependence/
83	“opioid use”.mp.
84	“opioid use disorder*”.mp.
85	Opioid related disorder*.mp.
86	(opioid adj2 disorder*).mp.
87	(opioid adj2 addict*).mp.
88	(opioid adj2 abuse).mp.
89	(opioid adj2 dependen*).mp.
90	or/79-89
91	37 and 78 and 90
92	39 or 91
93	limit 92 to (english or french)

#### Electronic databases

We will search MEDLINE Epub Ahead of Print, In-Process & Other Non-Indexed Citations, Ovid MEDLINE(R) Daily and Ovid MEDLINE(R), EMBASE, PsycINFO, CINAHL Plus with Full Text, and Cochrane Central Register of Controlled Trials.

#### Gray literature and search strategy

We will also include gray literature. As noted by several researchers, systematically searching for gray literature is a challenging process [64–66]. Using recommendations by Godin et al. [66], we have developed a preliminary list of gray literature sources identified to date, and the detailed gray literature plan will emerge from these and included peer-reviewed studies as they are identified. We will search theses/dissertation databases and repositories, gray literature databases, associations/societies, and community and government agency reports, as well as conference websites (Table 3).

**Table 3:** Preliminary gray literature resources

Resource type	Name	Description	URL
Dissertations	Proquest Dissertations and Theses Global	Global listing of dissertations and theses. Note, as open access archiving increases, fewer dissertations/theses are submitted to this resources	Subscription via University of Calgary
	British Library Ethos (e-theses online service)	Includes open access dissertations/theses from the UK	http://ethos.bl.uk/Home.do;jsessionid=876E1C64D231F26762053E178FFD5945
	Australasian Digital Theses (ADT) database via TROVE	Includes open access dissertations/theses from Australia	https://trove.nla.gov.au/book/result?l-australian=y&l-format=Thesis&q=&sortby=dateDesc
	Theses Canada Portal	Canadian dissertations and theses. Includes open access. Note, not updated since 2016	https://www.bac-lac.gc.ca/eng/services/theses/Pages/theses-canada.aspx
Databases	Canadian Electronic Library	Includes public documents from Canadian provincial and federal government and community agencies.	Subscription via University of Calgary
	OpenGrey	Open access to gray literature (paper) produced in Europe	http://www.opengrey.eu/
Addiction associations / societies	American Society of Addiction Medicine	A professional medical society representing physicians, clinicians and associated professionals in the field of addiction medicine	https://www.asam.org/
	Canadian Society of Addiction Medicine	Provides scientific and medical information about Addiction, for professionals and the general public	https://www.csam-smca.org/
	International Society of Addiction Medicine	Worldwide association for physicians working in addiction	http://www.isamweb.org/
	American Academy of Addiction Psychiatry	Professional organization focused on addiction psychiatry research and clinical treatment	https://www.aaap.org/
	American Association for the Treatment of Opioid Dependence	Works with US federal and state agency officials concerning opioid treatment policy	http://www.aatod.org/
	Center for Addiction and Mental Health	Canadian – Toronto - Canada’s largest mental health and addiction teaching hospital	https://www.camh.ca/
Other associations	Institute for Clinical and Economic Review	Independent and non-partisan research organization that objectively evaluates the clinical and economic value of prescription drugs, medical tests, and other health care and health care delivery innovations	https://icer-review.org/
	Federation of State Medical Boards	Supports the US states medical boards	https://www.fsmb.org/
	American Psychiatric Association	US psychiatric association	https://www.psychiatry.org/
	American Psychological Association	US psychologists association	http://www.apa.org/
Government	National Institute on Drug Abuse (US)	US government resource that advances science on the causes and consequences of drug use and addiction	https://www.drugabuse.gov/
	Substance Abuse and Mental Health Services Administration (US)	US agency that leads public health efforts to advance the behavioral health	https://www.samhsa.gov/
	Canadian Centre on Substance Use and Addiction	Created by the Canadian government to provide leadership addressing substance abuse	https://www.ccsa.ca

#### Other data sources

Our search will be supplemented by hand reviewing bibliographies of included articles and articles gathered from the authors’ personal files. The research team will also contact existing stakeholder groups (Canadian Research Initiative in Substance Misuse [CRISM] and the Canadian Society of Addiction Medicine [CSAM]) with expertise in OAT to identify relevant existing or emerging literature on this topic.

### Study selection

We will download search findings into Endnote, systematically de-duplicated, and create a merged library. The library will be exported into a draft MS Excel screening tool for title-abstract (level 1 [L1]) and full-text (level 2 [L2]) screening. The MS Excel screening tool will be piloted using 1–2 articles and finalized prior to using it. Two investigators (LLN, JCM) will calibrate screening by independently reviewing a 5% random sample of citations. Inter-rater agreement (kappa) [40] will be calculated and disagreements discussed to consensus for both level 1 and level 2 screening. If the kappa is < 0.60, study eligibility criteria will be revisited to focus on the screening criteria, and subsequent rounds of independent duplicate calibration will be undertaken until the desired kappa is achieved between investigators. Feedback and edits made to the screening tool during calibration will be discussed to consensus and the tool finalized.

#### Title and abstract [level 1 (L1)] screening

Once calibration is confirmed, the same two investigators (LLN, JCM) will review the title and abstract of all citations the search generates using the predetermined inclusion and exclusion criteria. The same two co-investigators (LLN, JCM) will calibrate screening of full-text articles independently and in duplicate. Studies meeting L1 inclusion-exclusion criteria will be submitted to full-text review (L2).

#### Full-text review [level 2 (L2) screening] and data extraction

We will retrieve full-text versions of all studies meeting the L1 inclusion-exclusion criteria for an in-depth review to confirm inclusion or record reasons for exclusion. In the manner described above, we will calibrate screening on a random sample (5%) of full-text articles in a similar fashion to level one, independently and in duplicate, by the same two co-investigators (LLN, JCM). Calibration will proceed until the concordance (kappa) between abstractors is *k* ≥ 0.60.

### Methodological quality and risk of bias

Two researchers (LLN, JCM) will assess, independently and in duplicate, the quality of included studies [70] using the Joanna Briggs Institute (JBI) Critical Appraisal Checklist for Qualitative Research, [71] which focuses on congruity within studies and is highly coherent. Risk of bias and validity of results in studies reporting quantitative data, will be assessed using Joanna Briggs Institute (JBI) Critical Appraisal Tools, to be determined by specific study design [72]. Investigators will choose 1–2 full-text studies and pilot the methodological quality assessment process to calibrate prior to conducting quality assessments for all included studies. Investigators will discuss discrepancies to resolution or refer to a third investigator (KJM) for a final decision. If the team determines the study is significantly flawed, the research team will discuss extreme scores and studies excluded to avoid inappropriate statistical and/or conceptual influence.

### Data extraction and analysis

We will use the Framework Analytic Method [74] as the primary method of data analysis to combine deductive and inductive qualitative and quantitative analysis (led by LLN, JCM, with KJM supporting). Ritchie and Spencer [74] designed this analytic method for use in applied settings and as a way to explore and/or inform social and public policy and programming. The framework approach also aligns well with systematic review methods and process, allowing the data to be identified, assessed, reduced, and summarized. Its advantage is that it provides a level analytic field within with which the team can consider evidence from multiple sources and theoretical origins (qualitative, quantitative, deductive, inductive). This approach helps guide the current research and enables the team to gather and consider findings and their implications within the proposed study in greater detail. This approach is useful in multi-disciplinary teams where familiarity with qualitative methodologies may be discrepant yet led by experienced qualitative researchers.

As detailed in the “Background” section above, we will use an a priori identified framework, a modified combination of the TDF and CFIR frameworks. Each framework has published template code books with definitions of their respective domains and subdomains [33, 35]. During L2 screening and full-text review, LLN and JCM will become familiar with the data, identify and extract data of interest relating to primary care prescribers’ perceptions of determinants of OAT prescribing, and study demographics as outlined in Table 1. We will tabulate extracted data into an Excel spreadsheet and indicate for each data element whether it is a direct participant quote or analyzed data (e.g., results of thematic analysis or statistical analysis). Calibrating data extraction between the two researchers will be conducted as in level 1 screening, with subsequent rounds of calibration if kappa is < 0.60.

Two researchers (LLN, JCM) will conduct a pilot coding exercise to practice applying the a priori TDF-CFIR framework codes to extracted data. The researchers will identify the extracted data as barriers or facilitators and categorically code each, as appropriate, into the framework [32, 33, 35] (see Table 1) using pre-determined code definitions and a coding structure into a qualitative analytic software program (NVivo [67]). For example, fear of patients diverting their OAT (giving or selling it to others) is an identified barrier for some primary care clinicians considering prescribing; this would be coded in the TDF domain “Beliefs about consequences.” LLN and JCM will independently code three randomly selected studies for pilot coding, and discrepancies will be resolved through consensus-finding or reviewed with a third researcher (KJM).

After the initial pilot coding exercise, the same two researchers (LLN, JCM) will code (“index”) all of the extracted data into the modified combined TDF-CFIR framework domains and subdomains. Code definitions will be strictly applied, and double codes will be applied only when necessary. Any barriers, facilitators, or strategies arising that do not align with the a priori framework will be cataloged and analyzed inductively to generate appropriate codes; these will be captured thematically using a team-agreed category name and definition.

After initial coding, the data extracted from the remaining studies and associated extracted data will be split between the coders, as has been done in other framework analysis studies [68]. After coding five different studies, both researchers (LLN, JCM) will independently code the same sixth study and meet to ensure coding consistency and discuss discrepancies. This will continue until data extracted from all included studies are coded. This approach will promote consistent application of the codes, testing, and possible refining of the code definitions to reflect the extracted data context and help develop a potential subset of domains/subdomains that are most relevant to the studies included. Studies with multiple reports will be cataloged and examined. If they overlap, their data will be extracted but combined. Once full-text review has been completed, a dated library of retained articles will be generated in Endnote and the search, study tools, library, and link to the published study protocol will be accessible on the Open Science Framework [69, 70].

Once deductive and inductive coding (indexing) has been completed, investigators will review all coded data within domains and subdomains to identify themes and possible relations among them (e.g., hierarchical or overlapping concepts). This will involve looking within and across domains, as well as within and across included studies; in keeping with framework analysis, this sorting and synthesizing process will benefit from using matrices generated by NVivo. This will allow three researchers (LN, JCM, KJM) as a group to view all coded data and their domains at once and by consensus; the team will inductively identify and describe patterns of similar views in the coded data relating to barriers and facilitators to primary care OAT prescribing.

We will also review each domain and associated data to determine quantitatively and qualitatively those likely to have the greatest influence on primary care prescribing of OAT. The former will involve frequency counts of the number of data elements coded in a particular domain, subdomain, or inductively generated category, as well as the number of studies from which extracted data was coded to a particular domain/category. We will also look for evidence of the importance of each coded perceived barrier, facilitator, or strategy based on statements by study participants (primary data) or findings reported by the study authors. We will generate “heat maps” with NVivo’s data density tool which uses a warm-to-cool color spectrum to represent frequency counts of the coded data. This gives a sense of where the “conversation” sits with regard to the coding framework, providing a visual representation of the synthesis of the findings.

We will examine the extracted strategies as a group to determine whether they can be combined. Finally, where it is feasible, the team will attempt to map identified effective strategies backwards onto precursor barrier-facilitator categories using the Theoretical Domains Framework [75], COM-B, and Behaviour Change Wheel [38, 75] to help enhance readiness of the data for subsequent use in modifying the intervention and/or study design.

Based on preliminary searching, we anticipate both quantitative and qualitative studies describing barriers, facilitators, and intervention strategies will emerge in the literature; however, due to heterogeneity and outcomes measures, we do not anticipate being able to conduct a meta-analysis. Should multiple studies with similar characteristics (context, outcomes measures) arise in the review, the team will explore the possibility of meta-analysis.

**Fig. 1:**
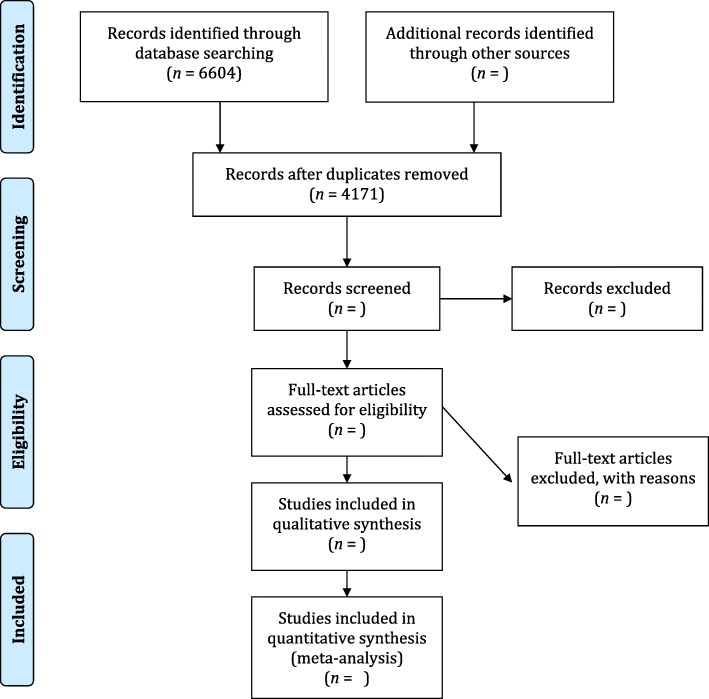
Draft flowchart for selection of studies

We will report study capture and flow using a PRISMA diagram (Fig. 1) [42]. Study characteristics will be presented in tabular format using descriptive statistics (frequencies and categories [*n*/%]). Barriers and facilitators will be reported similarly and using a heat map to quantify both the frequency and type of codes arising across included studies. We will provide coded data in the final systematic review report to facilitate its uptake and use by theorists and other researchers in the design of intervention strategies aimed at enhancing opioid agonist prescribing in primary care.

We will group interventions by type and summarize them narratively, if a sufficient number of eligible studies exist. Extracted strategies will be presented in tabular format, according to themes, as appropriate, and if meta-analysis is possible, appropriate statistics and forest plots will be used to report findings. We will make every effort to describe intervention strategies using key elements of interventions outlined by the TIDieR checklist; however, we anticipate missing data in this area given the documented lack of detail in most intervention reports [76]. If it is possible to map intervention strategies back to precursors, we will collate this information and present it in tabular format in the final report. We will report methodological quality in tables, according to tool results, and include the findings in an appendix in the final manuscript and note selective reporting within studies. All amendments to the protocol will be itemized as part of the study audit trail and reported on in the final manuscript.

## Discussion

Through this review, we will systematically identify and synthesize the evidence base related to barriers and facilitators to OAT prescribing perceived by primary care providers. By deductively coding the evidence using a modified combination of the TDF and CFIR frameworks, as well as applying inductive methods to identify unanticipated factors, the results of this review will provide a broad conceptual understanding of the determinants of implementing OAT prescribing that could help inform the evaluation of current interventions or the rapid development of new, evidence-based and theory-driven innovations promoting primary care prescribing to improve access to OAT in the current opioid public health crisis in North America.

A potential limitation of this study is that we are relying on data reported and interpreted by others. There is an associated potential risk of “reporting bias” with primary authors presenting selected data to support their project aims and excluding data relevant to this systematic review. In spite of these potential limitations, we propose that the enhanced analytic effort of the available primary literature proposed in this review recognizes both the need to comprehensively document and evaluate the literature pertaining to barriers, facilitators, and effective interventions for OAT prescribing in primary care settings and the need to consider how context may influence the same.

In closing, the intent of this systematic review is to generate findings and facilitate the design of high-quality, theory-driven, evidence-based intervention strategies to encourage primary care providers to prescribe opioid agonists. It is hoped the study results will have immediate and sustained relevance to individual practitioners, as well as educators, system planners, and policymakers.

## Additional file

Additional file 1: PRISMA-P Checklist. (DOCX 30 kb)

## Data Availability

Data sharing is not applicable to this article as no datasets were generated or analyzed during the current study. Materials, including final search strategy, Endnote library, and data extraction tools, will be available on the Open Science Framework (https://osf.io/).

## Abbreviations

CFIR: Consolidated Framework for Implementation Research [35]
CIHR: Canadian Institutes of Health Research
COM-B: Capability-Opportunity-Motivation-Behaviour Model [72]
CRD: The Centre of Reviews and Dissemination (CRD) guide [41]
ENTREQ: Enhancing Transparency in Reporting the Synthesis of Qualitative Research (ENTREQ) reporting guidelines [44]
JBI: Joanna Briggs Institute
MEDLINE©: Electronic database of medical journal citations and abstracts for global biomedical literature
MeSH: Medical Subject Headings
MS: Microsoft
NVIVO: Qualitative Analysis Software Package by QSR International [64]
OAT: Opioid agonist therapy
OUD: Opioid use disorder
PI: Principle investigator
PRESS: Peer Review of Electronic Search Strategies [61–63]
PRISMA: Preferred Reporting Items for Systematic Review and Meta-Analysis Statement [42]
PRISMA-P: Preferred Reporting Items for Systematic Review and Meta-Analysis Statement-Protocol Extension [43]
PROSPERO: International database of prospectively registered systematic reviews in health and social care
STARLITE: Sampling Strategy-Type of Study-Approaches-Range of Years-Limits-Inclusion and Exclusions-Terms Used-Electronic Source Search Reporting Standards [39]
TDF: Theoretical Domains Framework [32–34]
TIDieR: Template for Intervention Description and Replication checklist and guide [73]
US FDA: United States Food and Drug Administration

## References

[1] Nielsen S, Larance B, Degenhardt L, Gowing L, Kehler C, Lintzeris N (2016). Opioid agonist treatment for pharmaceutical opiod dependent people. Cochrane Database Syst Rev..

[2] Mattick RP, Breen C, Kimber J, Davoli M. Buprenorphine maintenance versus placebo or methadone maintenance for opioid dependence. Cochrane Database Syst Rev. 2014;(2):CD002207. 10.1002/14651858.CD002207.pub4. 10.1002/14651858.CD002207.pub4 PMC10617756 24500948

[3] Fiellin DA, Friedland GH, Gourevitch MN. Opioid dependence: rationale for and efficacy of existing and new treatments. Clin Infect Dis 43(Suppl 4):S173–S177, referenced by Torrens M, Fonseca F, Galindo L, and Farre M. Opioid Addiction: short- and long-acting opioids. Chapter 28 In El-Guebaly, Nady., Giuseppe. Carrà, and Marc. Galanter (Eds). Textbook of Addiction Treatment: International Perspectives. 2015. Web. 2006. 10.1086/508180 17109303

[4] Sordo L, Barrio G, Bravo MJ, Indave BI, Degenhardt L, Wiessing L, Ferri M, Pastor-Barriuso R (2017). Mortality risk during and after opioid substitution treatment: systematic review and meta-analysis of cohort studies. Br Med J..

[5] Mattick RP, Breen C, Kimber J, Davoli M (2009). Methadone maintenance therapy versus no opioid replacement therapy for opioid dependence. Cochrane Database Syst Rev..

[6] Mattick RP, Breen C, Kimber J, Davoli M (2014). Buprenorphine maintenance versus placebo or methadone maintenance for opioid dependence. Cochrane Database Syst Rev..

[7] Brogly SB, Saia KA, Walley AY, Du HM, Sebastiani P (2014). Prenatal buprenorphine versus methadone exposure and neonatal outcomes: systematic review and meta-analysis. Am J Epidemiol..

[8] Zedler BK, Mann AL, Kim MM, Amick HR, Joyce AR, Murrelle EL, Jones HE (2016). Buprenorphine compared with methadone to treat pregnant women with opioid use disorder: a systematic review and meta-analysis of safety in the mother, fetus and child. Addiction.

[9] Parmenter J, Mitchell C, Keen J, Oliver P, Rowse G, Neligan I, Keil C, Mathers N (2013). Predicting biopsychosocial outcomes for heroin users in primary care treatment: a prospective longitudinal cohort study. Br J Gen Pract..

[10] Barry CL, McGinty EE, Pescosolido B, Goldman HH (2014). Stigma, discrimination, treatment effectiveness and policy support: comparing public views about drug addiction with mental illness. Psychiatr Serv..

[11] Redko C, Rapp RC, Carlson RG (2008). Waiting time as a barrier to treatment entry: perceptions of substance users. J Drug Issues..

[12] Woo J, Bhalerao A, Bawor M, Bhatt M, Dennis B, Mouravska N, Zielinski L, Samaan Z. ‘Don’t judge a book by its cover’: a qualitative study of methadone patients’ experiences of stigma. Substance Abuse: Research and Treatment. (2017). 10.1177/1178221816685087. 10.1177/1178221816685087 PMC5398333 28469424

[13] Hirchak KA, Murphy SM (2017). Assessing differences in the availability of opioid addiction therapy options: rural versus urban and American Indian reservation versus nonreservation. J Rural Health.

[14] Sharma A, Kelly S, Mitchell M, Gryczynski S, O’Grady G, Schwartz J (2017). Update on barriers to pharmacotherapy for opioid use disorders. Curr Psychiatr Rep.

[15] Fonseca J, Chang A, Chang F (2018). Perceived barriers and facilitators to providing methadone maintenance treatment among rural community pharmacists in Southwestern Ontario. J Rural Health.

[16] Ford R, Bammer G, Becker N (2008). The determinants of nurses’ therapeutic attitude to patients who use illicit drugs and implications for workforce development. J Clin Nurs..

[17] Howard MO, Chung SS (2000). Nurses’ attitudes toward substance misusers. I. Surveys. Subst Use Misuse.

[18] Johansson L, Wiklund-Gustin L (2016). The multifaceted vigilance—nurses’ experiences of caring ncounters with patients suffering from substance use disorder. Scand J Caring Sci..

[19] Morozova O, Dvoriak S, Pykalo I, Altice FL (2017). Primary healthcare-based integrated care with opioid agonist treatment: first experience from Ukraine. Drug Alcohol Depend..

[20] Livingston J, Adams E, Jordan M, Macmillan Z, Hering R (2018). Primary care physicians’ views about prescribing methadone to treat opioid use disorder. Subs Use Misuse.

[21] Mintzer IL, Eisenberg MD, Terra M, MacVane C, Himmelstein DU, Woolhandler S (2007). Treating opioid addiction with buprenorphine-naloxone in community-based primary care settings. Ann Fam Med..

[22] Alford DP, LaBelle CT, Kretsch N, Bergeron A, Winter M, Botticelli M, Samet JH (2011). Collaborative care of opioid-addicted patients in primary care using buprehorphine: five year experience. Arch Intern Med..

[23] Guan Q, Khuu W, Spithoff S, Kiran T, Kahan M, Tadrous M, Martins D, Leece P, Gomes T (2017). Patterns of physician prescribing for opioid maintenance treatement in Ontario, Canada in 2014. Drug Alcohol Depend..

[24] Fraeyman J, Symons L, Van Roven P, Van Hal G, Peremans L (2016). How to overcome hurdles in opiate substitution treatment? A qualitative study with general practitioners in Belguim. Eur J Gen Pract..

[25] McMurphy S, Shea J, Switzer J, Turner BJ (2006). Clinic-based treatment for opioid dependence: a qualitative inquiry. Am J Health Behav..

[26] Barry DT, Irwin KS, Jones ES, Becker WC, Tetrault JM, Sullivan LE, Hansen H, O'Connor P, Schottenfeld RS, Fiellin DA (2008). Integrating buprenorphine treatment into office-based practice: a qualitative study. J Gen Intern Med..

[27] Becker WC, Fiellin DA (2005). Provider satisfaction with office-based treatment of opioid dependence: a systematic review. Subst Abuse..

[28] Islam MM, Topp L, Day CA, Dawson A, Conigrave KM (2012). The accessibility, acceptability, health impact and cost implications of primary healthcare outlets that target injecting drug users: a narrative synthesis of literature. Int J Drug Policy..

[29] Islam MM, Topp L, Day CA, Dawson A, Conigrave KM (2012). Primary healthcare outlets that target injecting drug users: opportunity to make services accessible and acceptable to the target group. Int J Drug Policy..

[30] Ford C (2012). Primary care is the best place to care for drug users. Int J Drug Policy..

[31] Grol R, Grimshaw JG (2003). From best evidence to best practice: effective implementation of change in patients’ care. Lancet..

[32] Michie S, Johnston M, Francis J, Hardeman W, Eccles M (2008). From theory to intervention: mapping theoretically derived behavioural determinants to behaviour change techniques. Applied Psychol..

[33] Michie S (2005). Making psychological theory useful for implementing evidence based practice: a consensus approach. Qual Saf Health Care..

[34] Cane J, O’Connor D, Michie S (2012). Validation of the theoretical domains framework for use in behaviour change and implementation research. Implement Sci..

[35] Damschroder LJ, Aron DC, Keith RE, Kirsh SR, Alexander JA, Lowery JC (2009). Fostering implementation of health services research findings into practice: a consolidated framework for advancing implementation science. Implement Sci..

[36] Birken SA, Powell BJ, Presseau J, Kirk MA, Lorencatto F, Gould NJ, Shea CM, Weiner BJ, Francis JJ, Yu Y, Haines E, Damschroder LJ (2017). Combined use of the Consolidated Framework for Implementation Research (CFIR) and the Theoretical Domains Framework (TDF): a systematic review. Implement Sci..

[37] Graham-Rowe E, Lorencatto F, Lawrenson JG, Burr J, Grimshaw JG, Ivers NM, Peto T, Bunce C, Francis JJ (2016). WIDeR-EyeS Project Team. Barriers and enablers to diabetic retinopahty screening attendance: protocol for a systematic review. Syst Rev..

[38] Michie S, Atkins L, West R (2014). The behaviour change wheel: a guide to designing interventions.

[39] Booth A (2006). “Brimful of STARLITE”: toward standards for reporting literature searches. J Med Lib Assoc.

[40] Higgins JP, Green S. Cochrane handbook for systematic reviews of interventions. 2011. http://handbook.cochrane.org. Accessed 5 Nov 2015.

[41] Centre for Reviews and Dissemination (CRD). Systematic reviews: CRD’s guidance for undertaking reviews in health care. Layerthorpe, York: University of York; 2009. https://www.york.ac.uk/media/crd/Systematic_Reviews.pdf. Accessed 5 Nov 2015.

[42] Moher D, Liberati A, Tetzlaff J, Altman D, PRISMA Group (2009). Preferred reporting items for systematic reviews and meta-analyses: the PRISMA statement. Annals Intern Med.

[43] Moher D, Shamseer L, Clarke M, Ghersi D, Liberati A, Petticrew M, Shekelle P, Stewart LA, PRISMA-P Group (2015). Preferred reporting items for systematic review and meta-analysis protocols (PRISMA-P) 2015 statement. Syst Rev.

[44] Tong A, Flemming K, McInnes E, Oliver S, Craig J (2012). Enhancing transparency in reporting the synthesis of qualitative research: ENTREQ. BMC Med Res Methodol..

[45] Straus S, Tetroe J, Graham ID, Straus S, Tetroe J, Graham ID (2013). Introduction knowledge translation: what it is and what it isn’t. Knowledge translation in health care: moving from evidence to practice.

[46] Graham I, Logan J, Harrison MB, Straus SE, Tetroe J, Caswell W, Robinson N (2006). Lost in knowledge translation: time for a map?. J Contin Educ Health Prof..

[47] Graham ID, Tetroe J, KT Theories Research Group (2007). Some theoretical underpinnings of knowledge translation. Acad Emerg Med..

[48] World Health Organization (1978). Alma Ata Declaration. Primary health care, health for all.

[49] Donaldson MS, Yordy KD, Lohr KN, Vanselow NA, Institute of Medicine (US) Committee on Primary Care (1996). Primary care: America’s health in a new era.

[50] Davis K, Schoenbaum MD, Audet A (2005). A 2020 vision of patient-centered primary care. J Gen Intern Med..

[51] The College of Family Physicians of Canada (2000). Primary care and family medicine in Canada: a prescription for renewal.

[52] Fiellin DA, Pantalon MV, Pakes JP, O’Connor PG, Chawarski MC, Schottenfield RS (2002). Treatment of opiate dependence with buprenophine in primary care. Am J Drug Alcohol Abuse..

[53] O’Connor PG, Fiellin DA (2000). Pharmacologic treatment of heroin-dependent patients. Ann Intern Med..

[54] Ferner RE, Daniels AM (2003). Office-based treatment of opiod-dependent patients. N Engl J Med..

[55] Institute of Medicine (1995). Executive summary: federal regulation of methadone treatment.

[56] US Food and Drug Administration. Drugs@FDA: FDA approved drug products. 2018. https://www.accessdata.fda.gov/scripts/cder/daf/index.cfm?event=reportsSearch.process&rptName=2&reportSelectMonth=10&reportSelectYear=2002&nav. Accessed 23 Apr 2018.

[57] Rambout L, Tashkandi M, Hopkins L, Tricco AC (2014). Self-reported barriers and facilitators to preventive human papillomavirus vaccination among adolescent girls and young women: a systematic review. Prev Med..

[58] Mrklas KJ, MacDonald S, Shea-Budgell MA, Bedingfield N, Ganshorn H, Glaze S, Bill L, Healy B, Healy C, Guichon J, Colquhoun A, Bell C, Richardson R, Henderson R, Kellner J, Barnabe C, Bednarcyzyk RA, Letendre A, Nelson GS. Barriers, supports and effective interventions for uptake of human papillomavirus and other vaccines within global and Canadian Indigenous peoples: a systematic review protocol. Syst Rev. 2018;7:1–20. 10.1186/s13643-018-0692-y PMC5833130 29499749

[59] Dooley Jessica, Asbridge Mark, Fraser John, Kirkland Susan (2012). Physicians’ attitudes towards office-based delivery of methadone maintenance therapy: results from a cross-sectional survey of Nova Scotia primary-care physicians. Harm Reduction Journal.

[60] Strang J, Manning V, Mayet S, Ridge G, Best D, Sheridan J (2007). Does prescribing for opiate addiction change after national guidelines? Methadone and buprehorphine prescribing to opiate addicts by general practitioners and hospital doctors in England, 1995-2005. Addiction..

[61] Sampson M, McGowan J, Cogo E, Grimshaw J, Moher D, Lefebvre C (2009). An evidence-based practice guideline for the peer review of electronic search straetgies. J Clin Epidemiol..

[62] McGowan J, Sampson M, Lefebvre C (2010). An evidence based checklist for the peer review of electronic search strategies (PRESS EBC). Evid Based Libr Inf Pract..

[63] McGowan J, Sampson M, Salzwedel D, Cogo E, Foerster V, Lefebvre C (2016). Guideline statement: PRESS peer review of electronic search strategies 2015 guideline statement. J Clin Epidemiol..

[64] Benzies KM, Premji S, Hayden KA, Serrett K (2006). State-of-the-evidence reviews: advantages and challenges of including grey literature. Worldviews Evid Based Nurs..

[65] Mahood Q, Van Eerd D, Irvin E (2014). Searching for grey literature for systematic reviews: challenges and benefits. Res Synth Methods.

[66] Godin K, Stapleton J, Kirkpatrick SI, Hanning RM, Leatherdale ST (2015). Applying systematic review search methods to the grey literature: a case study examining guidelines for school-based breakfast programs in Canada. Syst Rev..

[67] International Q (2014). NVivo10 for Windows.

[68] Keith R, Crosson J, O'Malley A, Cromp D (2017). Using the Consolidated Framework for Implementation Research (CFIR) to produce actionable findings: a rapid-cycle evaluation approach to improving implementation. Implementation Science.

[69] Foster ED, Deardorff A (2017). Open science framework (OSF). J Med Libr Assoc..

[70] Nixon L, Marlinga J, Hayden A, Mrklas KJ (2018). Barriers and faciliators to office-based opioid agonist therapy prescribing and effective interventions to increase provider prescribing: protocol for a systematic review.

[71] Hannes K, Lockwood C, Pearson A (2012). A comparative analysis of three online appraisal instruments’ ability to assess valididty in qualitative research. Qual Health Res..

[72] Joanna Briggs Institute (2017). The Joanna Briggs Institute critical appraisal tools for use in JBI systematic reviews: checklist for qualitative research.

[73] Joanna Briggs Institute. Joanna Briggs Institute - critical appraisal tools. 2017. http://joannabriggs.org/research/critical-appraisal-tools.html. Accessed 11 Apr 2018.

[74] Ritchie J, Spencer L, Bryman A, Burgess RG (1994). Qualitative data analysis for applied policy research. Analyzing Qualitative Data.

[75] Michie S, van Stralen M, West R (2011). The behaviour change wheel: a new method of characterising and designing behaviour change interventions. Implement Sci..

[76] Hoffman TC, Glasziou PP, Boutron I, Milne R, Perera R, Moher D, Altman D, Barbour V, Macdonald H, Johnston M, Lamb SE, Dixon-Woods M, McCulloch P, Wyatt JC, Chan A, Michie S (2014). Better reporting of interventions: template for intervention description and replication (TIDieR) checklist and guide. Br Med J..

